# Leopard seal diets in a rapidly warming polar region vary by year, season, sex, and body size

**DOI:** 10.1186/s12898-020-00300-y

**Published:** 2020-06-03

**Authors:** Douglas J. Krause, Michael E. Goebel, Carolyn M. Kurle

**Affiliations:** 1 Antarctic Ecosystem Research Division, NOAA Fisheries-Southwest Fisheries Science Center, 8901 La Jolla Shores Dr., La Jolla, CA USA; 2 Divsion of Biological Sciences, University of California San Diego, 9500 Gilman Dr., La Jolla, CA USA

**Keywords:** Stable isotope mixing model, Apex predator, Top down, Prey shift, Hydrurga leptonyx, Climate change, Animal-borne video

## Abstract

**Background:**

Resolving the preferred prey items and dietary proportions of leopard seals is central to understanding food-web dynamics in the rapidly-warming Antarctic Peninsula region. Previous studies have identified a wide range of prey items; however, due to anecdotal or otherwise limited information, leopard seal diets remain unresolved by seal sex, individual, body size, region, and season. Over the 2013, 2014, and 2017 field seasons we collected scat, tissue samples (red blood cells and plasma; n = 23) for stable isotope analyses, and previously-reported animal-borne video from 19 adult leopard seals foraging near mesopredator breeding colonies at Cape Shirreff, Livingston Island. We summarized a priori diet information from scat and video analysis and applied a three-isotope (*δ*^13^C, *δ*^15^N, *δ*^34^S), four-source (fish, fur seal, krill, penguin) Bayesian mixing model to examine temporal variability in both prey sources and leopard seal tissues.

**Results:**

The austral spring diets of males and females focused on Antarctic krill (31.7–38.0%), notothen fish (31.6–36.5%), and penguin (24.4–26.9%) and were consistent across all 3 years. Several lines of evidence suggest the transition to summer foraging was distinct for males and females. Female diets transitioned rapidly to higher *δ*^15^N values (+2.1‰), indicating increased consumption of penguin (29.5–46.2%) and energy-dense Antarctic fur seal pup (21.3–37.6%).

**Conclusions:**

The seasonal increase in leopard seal *δ*^15^N values, and thus fur seal in their diet, was predictably related to larger body size; it may also be forcing reductions to the largest Antarctic fur seal colony in the Antarctic Peninsula. Our ensemble sampling approach reduces historical biases in monitoring marine apex predator diets. Further, our results are necessary to best inform regional fisheries management planning.

## Background

Changes in the foraging behavior of large marine predators can fundamentally transform ecosystems through cascading predator–prey interactions [1–3]. The Southern Ocean supports one of the largest communities of endothermic predators in the world [4, 5], and many of those populations are fluctuating in a swiftly warming climate [6–8]. Leopard seals (*Hydrurga leptonyx*) are apex Antarctic predators capable of foraging across a range of trophic levels from mesopredators (e.g., penguins and seals) to fish and krill [9, 10]. Quantifying their effect on coastal ecosystems is dependent upon measuring their diet variability across demographic categories and over a variety of time scales. Focal studies of leopard seals have been difficult to conduct, however, leading to a poor understanding of their foraging ecology.

Reports on leopard seal diets using anecdotal data (e.g., reviewed by 11), and stomach contents [9, 12], scats [11, 13], and fatty acid analysis [14] indicate that leopard seals eat planktivorous krill and fish, as well as squid, seabirds, and seals. Krill stands out as a potentially key diet component, but is not consistently observed (e.g., [15]), and these studies generally employed biased techniques. For example, stomach-content and scat data from pinnipeds differentially represent recently consumed, and hard-shelled prey [16, 17] and are further biased by varying prey digestion rates [18]. Therefore, while leopard seal prey items have been identified, diets remain unresolved in terms of variation between seal sexes, and among individuals, age classes, regions, and seasons.

Multiyear behavioral studies utilizing focal observations, bio-loggers, and animal-borne cameras have shown evidence of specialization by individual leopard seals in prey selection, area use [19–21], and temporal foraging activity [22]. Individual foraging specialization is both taxonomically widespread and ecologically important [23]; however short observation periods (e.g., video, scat analysis) tend to overestimate individual specialization [24].

Stable isotope analyses (SIA) avoid some of these biases and are commonly used to study the trophic ecology of free ranging pinnipeds (e.g., [25–28]). Stable carbon (*δ*^13^C) and nitrogen (*δ*^15^N) isotopes are typically used in diet studies because they reflect the corresponding isotope values of the consumer’s prey field plus tissue-specific trophic discrimination factors which increase consumer isotope values through the processes of diet assimilation and excretion [29]. Stable sulfur (*δ*^34^S) isotopes also illustrate consumer diet shifts [30, 31], particularly for carnivores with high-protein diets [32].

Additionally, if isotope values for the consumer and prey items are known, stable isotope mixing models can quantitatively estimate the relative proportions of prey within consumer diets (e.g., [33, 34]). For example, an early isotope mixing model [35] illustrated seasonal variation and individual dietary separation for three leopard seals in East Antarctica [36]. However, these earlier models were unable to incorporate the variance of isotopic measurements which can have dramatic effects on dietary estimates [37]. Recently, Bayesian stable isotope mixing models explicitly characterize uncertainties around the isotopic measurements of consumer tissues, the trophic discrimination factors [38], and prey sources [39–41] to ensure that dietary proportions are reported with associated uncertainty.

A further advantage of SIA over conventional diet observations is that consumer tissues assimilate digested diet components over time scales that vary with tissue type. The corresponding time frame depends on the protein turnover rate of the sampled tissue. Therefore, temporal changes in diet can be detected by aligning the time scale of consumer tissue turnover rates with potential diet shifts [42–46]. Several studies have established turnover rates for various pinniped tissue types (e.g., [47, 48]). For example, isotope values from blood plasma and red blood cells provide dietary information on the order of approximately 1 week to 1 month, respectively, prior to pinniped tissue collection [49, 50].

There are limitations to using SIA for quantifying consumer diets, particularly when there is overlap in the stable isotope values from prey resources [51]. Historically, the greatest dietary resolution has been derived from studies which combined SIA and more traditional field data collection (e.g., observations, gut contents, and fecal samples) [52–54] as these methods can add a priori or a posteriori information to models to help ensure all prey sources are identified, which is a basic assumption of stable isotope mixing models [37]. Finally, the incorporation of additional stable isotopes (e.g., *δ*^34^S) to the more traditionally utilized *δ*^13^C and *δ*^15^N values can provide greater resolution for Bayesian mixing models when isotope values from potential prey overlap [55].

Describing leopard seal diet variability by individual, sex, and mass over relevant time scales is central to understanding their responses to environmental change [56], and the potential top-down effects they may impose on Antarctic ecosystems in a rapidly changing region [57, 58]. Therefore, the objectives of our study were to: (1) identify leopard seal prey sources (a priori) via analysis of beach-collected seal scats and animal-borne video of seal foraging behavior, (2) estimate their trophic position and dietary proportions using the *δ*^13^C, *δ*^15^N, and *δ*^34^S values from prey sources and leopard seal tissues within a Bayesian stable isotope mixing model, and (3) assess temporal and individual variation in seal diets by comparing red blood cell (RBCs) and plasma SI signatures sampled within and between years.

## Methods

### Study site

**Fig. 1:**
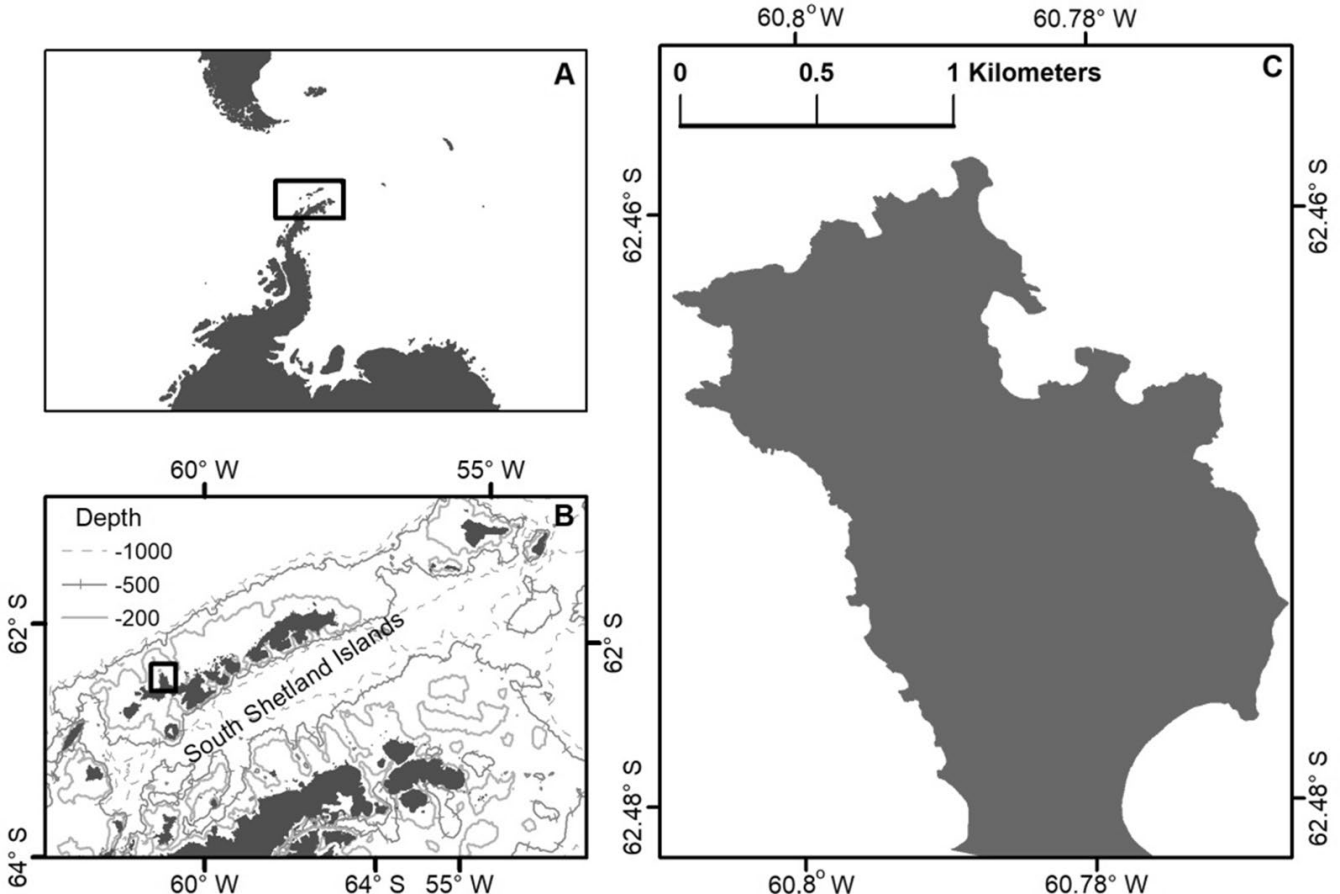
The U.S. AMLR long-term ecosystem monitoring site at Cape Shirreff, Livingston Island, Antarctic Peninsula

We conducted field studies within the U.S. Antarctic Marine Living Resources (AMLR) Program research area at Cape Shirreff on the north shore of Livingston Island, Antarctic Peninsula (Fig. 1, 62.47° S, 60.77° W). Since the late 1990s, adult female leopard seals have been hauling-out on land annually between late-December and May, with peak numbers in January and February, near mesopredator breeding colonies at Cape Shirreff. Reduced sea ice tends to concentrate leopard seals [59, 60]; therefore, the loss of sea ice near Cape Shirreff may have further increased leopard seal density by limiting available haul-outs to coastal beaches. Since 2010, leopard seal densities have increased by two orders of magnitude (> 20 seals/nautical mile^2^, [21]) over those reported by previous surveys in the region [58, 61]. Predation by leopard seals on breeding penguins (gentoo (*Pygoscelis papua*) and chinstrap (*Pygoscelis antarcticus*)) and Antarctic fur seal pups (*Arctocephalus gazella*) is common between December and March [21, 62], but the contributions of these mesopredators to leopard seal diets has been difficult to quantify.

### Stable isotope sampling: consumer

We selected and chemically immobilized [22, 63] healthy adult female (*n* = 17) and male (*n* = 2) leopard seals for blood collection during the course of the 2013 (*n* = 9), 2014 (*n* = 10), and 2017 (*n* = 4) field seasons. Field seasons were conducted between October 10 and March 16 each year. We sampled four of those same individuals in different years, providing 23 blood samples from 19 individuals (Table 1). Once sedated, we drew blood samples via a hypodermic needle into vacuumed, additive-free, blood-collection vials. We centrifuged the blood samples to separate plasma and RBC components, then stored them at − 20 °C. Over the three field seasons we also collected 46 scats and 25 visual prey-consumption observations of our study animals (Table 1).

**Table 1:** Individual identifications and summary foraging statistics for leopard seals sampled for red blood cells (RBC), plasma, and scat in 2013, 2014, and 2017 at Cape Shirreff, Livingston Island (23 samples from 19 individuals)

Seal id—Year	Sex	Deployment length (d)	Foraging trips (*n*)	Mass (kg)	Length (cm)	RBC and plasma collected (*n*)	Capture scat (*n*)	Opportunistic scat (*n*)	Visual observation (*n*)
12OR—2013	♀	6.98	3	446	305	2	–	3	–
36OR—2013	♀	3.5	2	437	307	2	1	–	6
37OR—2013	♀	–	–	409	301	1	–	3	–
62OR—2013	♀	12.10	4	422	293	2	–	–	–
70OR—2013	♀	–	–	421	287	1	1	–	–
71OR—2013	♀	6.12	3	460	285	2	–	1	1
394Y—2013	♀	4.69	2	416	285	2	1	1	–
406Y—2013	♀	9.12	4	498	312	2	1	–	3
422Y—2013	♀	0.86	1	416	293	2	1	–	3
09OR—2014	♀	8.53	6	414	301	2	–	4	2
16OR—2014	♀	4.32	1	494	311	2	1	–	–
18OR—2014	♀	7.53	6	494	296	2	2	3	–
37OR—2014	♀	4.28	3	406	301	2	–	–	–
58OR—2014	♀	–	–	409	301	1	1	–	–
63OR—2014	♀	8.55	9	432	305	2	–	2	–
84OR—2014	♀	8.46	8	371	284	2	1	–	–
397G—2014	♀	5.49	3	385	287	2	1	3	4
401Y—2014	♀	3.92	2	485	298	2	2	6	6
406Y—2014	♀	7.71	5	533	312	2	1	5	–
12OR—2017	♀	5.50	1	543	305	2	–	–	–
84OR—2017	♀	7.59	3	437	284	2	–	–	–
111OR—2017	♂	21.61	15	251	250	2	–	–	–
120OR—2017	♂	10.98	9	329	270	2	1	–	–
Mean (± SD)/Total		7.39 ± 4.29	90	431 ± 64	294 ± 15	43	15	31	25

Scats were collected during capture events or opportunistically. “Visual observations” are successful foraging events witnessed from shore or animal-borne video

We measured each animal for standard length and girth [64], and weighed each using a sling, tripod, hand winch, and a tensionometer (MSI-7300 Dyna-Link 2, capacity 1000 ± 0.5 kg). We assigned adult life stage based on standard lengths of > 2.5 m for males and > 2.7 m for females [65]. We collected scats deposited during the capture event in two-gallon plastic bags and froze them for later analysis. Of the 23 animals in our study, we deployed animal-borne cameras on seven (CRITTERCAM, [21]), and we recaptured 20 after ~ 1 week (7.39 ± 4.29 days) to recover instruments, re-sample the seals for blood (RBC/plasma), and re-weigh each individual. Upon capture completion, sedative-reversal pharmaceuticals were administered [63]. Each animal’s recovery was visually monitored until it reached a mobile state. After handling, all animals in this study were re-sighted in a healthy state at least once within 2 weeks of capture.

We thawed scats and rinsed them with fresh water through a series of stainless-steel sieves (range: 2.8–710 mm). We sorted hard prey parts (fish bone, otolith, fur seal bone, etc.) and identified them to species when possible. Additionally, field personnel patrolled study beaches daily and collected fresh scats (warm, no evidence of scavenging by shorebirds) from study animals. These were sorted without sieves, and components were identified to species when possible. In addition to visual observations from animal-borne cameras [21], several observers witnessed one study animal (36 OR) ambush hunting and consuming Antarctic fur seal pups on several occasions.

### Stable isotope sampling: prey sources

Based on a literature search from regional sites, scat analysis, and visual observations, we determined the potential prey field of leopard seals at Cape Shirreff to contain demersal fish (*notothen spp.*), Antarctic fur seal pups, gentoo (*Pygoscelis papua*) and chinstrap (*Pygoscelis antarcticus*) penguins, and Antarctic krill (*Euphausia superba*). Therefore, we collected samples of each prey type from Cape Shirreff during each of the 2013, 2014, and 2017 field seasons when possible. Because baseline stable isotope signals of potential prey sources can vary greatly over small spatial and temporal scales [37, 46, 66], we collected prey samples from Cape Shirreff concurrent with field sampling of leopard seal tissues.

We extracted Antarctic fur seal pup and penguin muscle tissue samples (~ 2 cm × 2 cm) with a scalpel from recently deceased animals that did not appear to be emaciated or obviously diseased. We opportunistically collected whole krill from the shoreline following large storms, and fish heads and bodies discarded by predatory shorebirds (Additional file 1: Table S1). Both fish and krill were the same species and body size of those identified in scats and video. Field personnel cleaned penguin muscle tissue with deionized water and dried the tissue at 60 °C for ≥ 24 h. All other prey samples were packed in plastic bags and frozen at − 20 °C until prepared for stable isotope analysis.

### Stable isotope sample preparation

We conducted all stable isotope sample preparation in the Kurle Lab at the University of California San Diego. We thawed all samples, rinsed with deionized water, freeze-dried for ≥ 24 h, and homogenized them with a metal spatula. We extracted lipids from animal tissues with C:N ratios ≥ 3.5 (fur seal and penguin muscle) according to Folch et al. [67] as modified by Sweeting et al. [68] and Post, Layman [69]. We placed each sample in an 18 ml glass tube, added 10 ml of petroleum ether, and sonicated the samples at 40 kHz for 10 min in a water bath warmed to 60 °C. We centrifuged the samples at 12,000*g* for 5 min, poured or pipetted off the petroleum ether, rinsed the samples with nanopure water, then sonicated the samples again with micro-pure water for 10 min. Sample vials were centrifuged again for 10 min and excess water removed. Finally, we transferred tissues to cryovials and dried them at 43 °C for 24–48 h.

### Stable isotope analysis

We packaged subsamples of dried, homogenized tissue (1.0 ± 0.5 mg for *δ*^13^C and *δ*^15^N, 4.0 ± 0.5 mg for *δ*^34^S) into 5 mm × 9 mm tin capsules for analysis. Personnel at the University of California Davis Stable Isotope Facility combusted the samples in a PDZ Europa ANCA-GSL elemental analyzer interfaced with a PDZ Europa 20–20 (*δ*^13^C and *δ*^15^N) or SerCon 20–22 (*δ*^34^S) isotope ratio mass spectrometer (Sercon Ltd., Cheshire, UK). They normalized the raw stable isotope values using laboratory standards calibrated against NIST Standard Reference Materials and calculated sample precision at 0.2‰, 0.3‰ and 0.4‰ for *δ*^13^C, *δ*^15^N, and *δ*^34^S respectively. The abundance of stable isotopes is expressed in notation according to the following equation:

1 $$\delta {\text{X }} = \frac{{{\text{R}}_{\text{sample}} - {\text{R}}_{\text{standard}} }}{{{\text{R}}_{\text{standard}} }}*1000$$

where X is ^13^C, ^15^N or ^34^S and R is the corresponding ratio of ^13^C/^12^C, ^15^N/^14^N or ^34^S/^32^S. The Rstandard value is set by PeeDee Belemnite for *δ*^13^C, atmospheric N_2_ for *δ*^15^N, or Vienna-Canyon Diablo Troilite for *δ*^34^S.

### Data analysis

We conducted statistical analyses using R [70]. Uncertainty in SIA analyses can be strongly related to the degree to which the stable isotope values from prey or prey categories are distinct from one another in multi-dimensional space [71]. We treated all *δ*^13^C–*δ*^15^N–*δ*^34^S three-dimensional (3D) “isospace” data as spatial [72] and tested for differences between groups of isotopic data using either a K nearest-neighbors randomization test (KNN) [73, 74] for 3D data, or Welch’s two-sample t tests for one-dimensional data. We tested the explanatory power of our model covariates (mass, sex, year) for each set of consumer isotope values using analysis of variance (ANOVA) and linear regression tests. All values are listed as mean ($\bar{X}$) ± standard deviation (SD), and all inferences were based on a significance level of P ≤ 0.05 unless otherwise indicated.

### A priori prey determinations

We summarized the relative proportions of prey consumed by leopard seals, as estimated from seal scats, in two ways: (1) we calculated the frequency of occurrence (F%) for each prey taxon as the percentage that a prey item was seen in all scats during that season as per Casaux et al. [13]. (2) Because of the partial and inconsistent consumption of prey items consumed by leopard seals, we lacked reliable prey hard parts (e.g., otoliths, jaw bones) needed to quantify the number or original volume of prey consumed. Therefore, as an alternative metric of prey importance, we estimated the volumetric proportion of each diet component in the overall scat composition. We used these scat data to support our a priori choice of prey sources for SIA analysis. We categorized results into “Scat Observations” and “Visual Observations” to reflect the fundamental differences in data collection. A detailed description of animal-borne video deployment and analysis can be found in Krause et al. [21].

### Grouping stable isotope data

Reducing the number of prey sources to the fewest ecologically-relevant groups is likely to improve the explanatory power of stable isotope mixing models [51]. Therefore, we grouped each prey type (a.k.a., “source”) into taxonomic categories, including only prey species for which we had evidence of consumption by leopard seals (see above). The “krill” and “fur seal” groups contained only a single species each, and we included the isotope values from all krill and fur seal samples in each group. We combined the stable isotope values from two species of demersal fishes, *Notothenia coriiceps* and *Trematomus newnesi,* and categorized them as “fish.” There was no support for isospace separation between gentoo and chinstrap penguins (KNN, P = 0.89), so we combined the isotope values from both species into one “penguin” prey category.

We collected representative prey samples from the “krill” and “fur seal” categories during all three seasons. We were only able to collect “fish” tissue during the 2014 and 2017 seasons, and “penguin” tissue during the 2013 and 2014 seasons. However, the *δ*^15^N values from “fish” and “penguin” corresponded closely with published values from locally conducted studies, Zamzow et al. [66] and Polito et al. [54] respectively, and there was no support for differences in the *δ*^13^C and *δ*^15^N values between years (KNN, P = 0.69). Therefore, we used source stable isotope values unique to the year of their collection for “krill” and “fur seal”, and we used the mean values from collected years for missing years for “fish” and “penguin.”

SIA mixing model theory dictates that each of the source groupings should be distinct in isospace [51, 73]. Therefore, we tested the prey source groupings within each field season using KNN analysis. The null hypothesis was that there was no spatial overlap between groups.

We grouped all leopard seal (a.k.a., “consumer”) isotopic data by tissue type, capture order (1st or 2nd capture), and year. During subsequent analyses, we matched each group with the corresponding source data (by year) and tissue-specific discrimination factor. For inter-annual or inter-tissue comparisons, we used only capture 1 data to maintain independence.

### Stable isotope trophic discrimination factors

Stable isotopes fractionate as prey tissues are broken down and assimilated into a consumer, generally leading to higher *δ*^13^C and *δ*^15^N values in consumer tissues compared to their prey [75]. Such differences are called trophic discrimination factors (TDFs), and are written with the notation Δ^13^C (and similarly for other stable isotopes). TDFs can vary by species and tissue type [75, 76] and are affected by a host of environmental and physiological variables ([37, 76] and references therein). To account for these variations, it is important to include TDFs that most closely reflect those of the consumer and tissues of interest as dietary estimates generated by the Bayesian stable isotope mixing models can vary widely with different TDFs [34, 77].

We used species and tissue specific TDFs for plasma and RBCs reported in the literature. We chose TDFs determined from captive feeding studies by taking the mean values of the seals most closely related to leopard seals, including: ringed seals (*Pusa hispida*), spotted seals (*Phoca largha*), and Hawaiian monk seals (*Monachus schauinslandi*). The resulting mean (± SD) TDF values for all prey sources in all seasons were: plasma Δ^13^C = 1.20 ± 0.14‰, Δ^15^N = 3.85 ± 0.49‰, and Δ^34^S = − 0.6 ± 0.15‰ and RBC Δ^13^C = 1.53 ± 0.10‰, Δ^15^N = 2.75 ± 0.44‰ [78], and Δ^34^S = − 0.6 ± 0.15‰ [32].

### Bayesian stable isotope mixing model (MixSIAR)

We used the Bayesian stable isotope mixing model MixSIAR [79, 80] to estimate leopard seal diet composition. We tested four-source (fish, fur seal, krill, penguin) models with combinations of two isotope (*δ*^13^C, *δ*^15^N=CN, *δ*^13^C, *δ*^34^S=CS, and *δ*^15^N, *δ*^34^S=NS), and three isotope (*δ*^13^C, *δ*^15^N, and *δ*^34^S=CNS) models. Candidate hierarchical models were created to evaluate each of our objective covariates: year, sex, mass, and individual (Seal ID). We applied year and seal ID as random effects, sex as a fixed effect, and mass as a continuous effect. To account for inter-annual shifts in *δ*^13^C values at the base of the food web, and because source data were indexed per year, we nested all model effects within year. For example, CNS ~ (1|Year/Seal ID) represents a three-isotope hierarchical model with seal ID nested within year.

We incorporated the stable isotope values (± SD) from the consumers (leopard seals), prey sources, and TDFs into MixSIAR for each year and tissue (plasma or RBC). Due to our low sample sizes (< 20), we modeled the relationship between source and consumer data using a “fully Bayesian” implementation [39]. Prior distributions for relative contributions of source data were Dirichlet, or “generalist,” distributions which are uninformative on the simplex, making all combinations of source data equally likely [80]. The models ran three Markov Chain Monte Carlo (MCMC) chains with 3 million iterations, a burn-in of 1.5 million, and were thinned by 500. MixSIAR reported results as mean, 1 SD, and credible intervals (CI) for each posterior density distribution per prey source. We tested model convergence using Gelman–Rubin and Geweke diagnostics as well as posterior density, trace, running means, and autocorrelation plots. We evaluated model performance using the Deviance Information Criterion (DIC) [81].

### Antarctic fur seal availability as potential prey

To provide context for model outputs, we calculated an index of Antarctic fur seal pup availability (as potential leopard seal prey) based on pup census and growth data. We tracked the pup survival of all known (identification-tagged) Antarctic fur seal females daily throughout the season because the survival rate of their pups is an index of survival for the entire pup population. We collected data on pup growth rates starting in early January (30 days after the date of median pupping). Over 4 intervals of 15 days, we randomly selected and weighed 100 fur seal pups. We multiplied the number of live pups by the average weekly pup mass for each of the first eight weeks of the year for 2013, 2014, and 2017.

Additionally, we established the number of locally foraging leopard seals by conducting a Cape Shirreff-wide, weekly phocid census during which we counted and reported all seals by species and life stage.

## Results

### A priori prey estimates from scat analysis and visual observations

The proportions of prey present in leopard seal diets based on analysis of seal scats and visual observations for 2013, 2014, and 2017 are summarized in Table 2. Fur seal, penguin, and krill remains were identifiable to species in scats, notably, fish were not. Leopard seals typically remove notothen fish heads during prey processing [21]; hence we found no otoliths or diagnostic fish parts in any scat. Therefore, we used identifications from video observations of fish consumption [21], all of which were notothen species (including *Notothenia coriiceps*) in concurrence with a previous regional report [13]. In 2013 and 2014 Antarctic fur seal was by far the largest prey source identified by frequency of occurrence (2013 = 100%; 2014 = 100%) followed by penguin (2013 = 30.8%; 2014—48.4%), fish (2013 = 23.1%; 2014 = 50.0%), and krill (2013 = 23.1%; 2014 = 0.0%). During 2017 the only scat collected from a study animal was from a male (120OR) and contained 100% krill.

**Table 2:** Frequency of occurrence (F%) and estimated percentage of total volume (V%) of prey items in leopard seal diets for 2013, 2014, and 2017 based on scat and visual observation data

Year	*n*	Scat observations						*n*	Visual observations			
		Fur seal		Penguin		Krill			Fur seal		Fish	
		F%	V%	F%	V%	F%	V%		F%	V%	F%	V%
2013	13	100	88.5	30.8	18.0	23.1	10.0	13	100	90.0	23.1	40.0
2014	31	100	77.5	48.4	50.6	0.0	0.0	12	100	70.0	50.0	90.0
2017	1	0.0	0.0	0.0	0.0	100	100	0	0.0	0.0	0.0	0.0

*n* is the number of scats collected or visual observations per individual in a given season. “Fur seal” was *Arctocephalus gazella*, “Penguin” was either *Pygoscelis papua* or *Pygoscelis antarcticus*, “Krill” was *Euphausia superba*, and “Fish” was either *Notothenia coriiceps* or unidentified nothen fish

### Stable isotope data

**Fig. 2:**
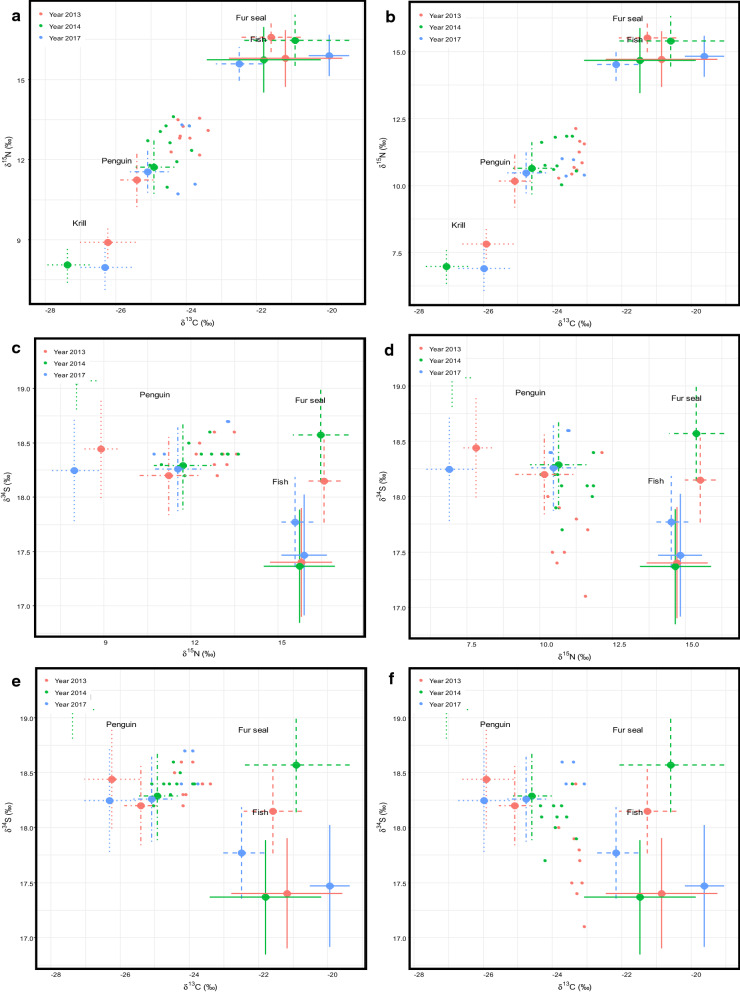
Paired isospace plots of the *δ*^13^C, *δ*^15^N, and *δ*^34^S values from leopard seal plasma (**a**, **c**, **e**) and RBCs (**b**, **d**, **f**) (*n *= 23, colored dots) plotted with concurrent prey stable isotope values (colored crosses: dotted = krill, dot-dash = penguin, dashed = fur seal, solid = fish) from 2013, 2014, and 2017. The prey sources were corrected using tissue specific (plasma or RBC) trophic discrimination factors

The *δ*^13^C, *δ*^15^N, and *δ*^34^S values for the four prey sources (Additional file 1: Table S1) were distinct in isospace, except for fur seal and fish (Table 3, Fig. 2a, b). Fish and fur seal overlap probabilities were mostly low (Table 3), so we elected to keep them as separate groups in the stable isotope mixing model. Isospace plots showing source ($\bar{X}$ ± SD) and individual leopard seal *δ*^13^C, *δ*^15^N, and *δ*^34^S values for each category of leopard seal tissue (plasma and RBC) from each year (2013, 2014, 2017) demonstrate that all consumer values fall within the range of the three isotope values from the prey sources (Fig. 2a–f), satisfying mixing model assumptions [37].

**Table 3:** P-values of K nearest-neighbors randomization tests (KNN) of proximity in isospace for the four leopard seal prey source groups from 2013, 2014, and 2017

	Fish	Fur seal	Krill
2013			
Fish	–		
Fur seal	0.142	–	
Krill	< 0.001	0.005	–
Penguin	< 0.001	0.007	< 0.001
2014			
Fish	–		
Fur seal	0.095	–	
Krill	< 0.001	< 0.001	–
Penguin	< 0.001	< 0.001	< 0.001
2017			
Fish	–		
Fur seal	0.011	–	
Krill	0.006	< 0.001	–
Penguin	0.001	< 0.001	< 0.001

K nearest-neighbor randomization tests showed no statistically significant spatial separation between the 1st and 2nd captures within those groups (KNN, P ≥ 0.99; Additional file 1: Table S2). We detected no trends or differences in the *δ*^13^C and *δ*^34^S values from leopard seal samples across years and tissues; however, there were some differences in their *δ*^15^N values. The *δ*^15^N values for all tissue samples across all years were slightly higher (≤ 0.8‰) for those animals sampled in capture 2 vs. 1, although this difference was not significant (Welch’s two-sample t-test; P = 0.23, t = − 1.2, df = 31).

**Fig. 3:**
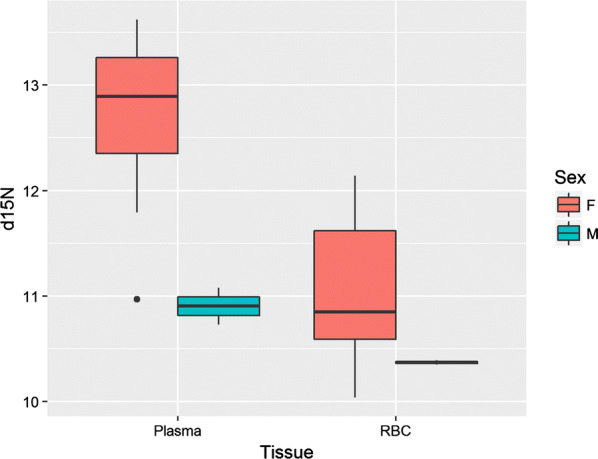
The *δ*^15^N values from all leopard seal plasma (*n *= 23) and RBCs (*n *= 23) collected in 2013, 2014, and 2017 and separated by sex, F = female (*n *= 21), M = male (*n *= 2). Boxes indicate median and upper and lower 75th and 25th percentiles; whiskers represent 10th and 90th percentiles, and dots represent values outside the 10th and 90th percentiles

**Fig. 4:**
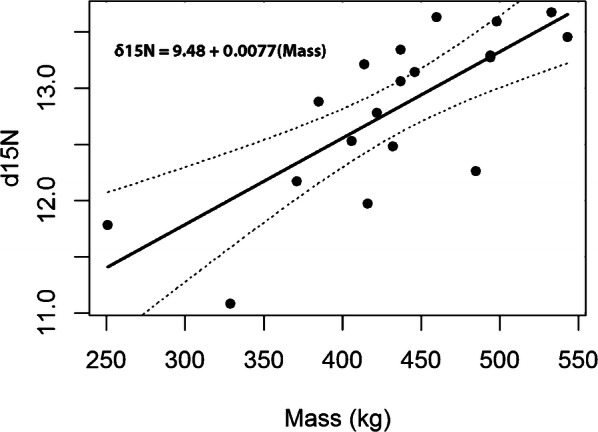
Linear relationship between the *δ*^15^N values from leopard seal plasma (*n *= 23) and leopard seal mass. Dotted lines indicate the 95% confidence intervals

Plasma tissues had higher *δ*^15^N values by 0.7 to 2.3‰ compared with those from RBCs for all seasons (*n* = 23, P < 0.001, t = 7.42, df = 22). Females exhibited a larger average difference than males (+2.1‰ and +0.5‰ respectively, Fig. 3). Finally, the *δ*^15^N values from leopard seal plasma had a positive relationship with seal body mass both with (Fig. 4; P < 0.001, Adjusted R^2^: 0.55) and without (P = 0.02, Adjusted R^2^: 0.31) males included; whereas the *δ*^13^C and *δ*^34^S values did not (P = 0.38, Adjusted R^2^: 0.05; and P = 0.94, Adjusted R^2^ < 0.01, respectively).

### Bayesian stable isotope mixing model (MixSIAR)

The stable isotope mixing models using only two isotopes (*δ*^13^C and *δ*^15^N, *δ*^13^C and *δ*^34^S, and *δ*^15^N and *δ*^34^S) failed to converge, even with extreme MCMC chain lengths (> 3 million) and yielded biologically unreasonable posterior estimates of diet due to a linear distribution of source values in isospace (e.g., Fig. 2a).

Conversely, the MCMC chains for the CNS models likely converged. The Gelman diagnostic for our isotope models incorporating three stable isotopes was < 1.05 for all variables, and the Geweke scores for all chains showed ≤ 5% of variables outside of ± 1.96. Additionally, posterior density plots by chain showed high correspondence, traceplots showed broad mixing through parameter space, running means converged over time, and autocorrelation plots showed a decrease with increasing iterations. For stable isotope values from both plasma and RBC tissue, the models with year alone as a random effect were the most parsimonious, and models with sex nested within year were comparably informative (Table 4).

**Table 4:** DIC model selection results for the *δ*^13^C, *δ*^15^N, *δ*^34^S stable isotope (CNS) Bayesian mixing models (MixSIAR) used to estimate diets of leopard seals based on stable isotope values from plasma and red blood cells (RBC) sampled from seals at Cape Shirreff, Livingston Island in 2013, 2014, and 2017 (*n* = 23)

Model	DIC	∆DIC	Median variance (σ)
CNS_plasma_ ~ (1|Year)	32.77379	–	σ_year_ = 0.772
CNS_plasma_ ~ Sex + (1|Year)	33.85499	1.0812	σ_year_ = 0.911 σ_Sex_ = 1.684
CNS_plasma_ ~ (1|Year/Seal ID)	40.84313	8.0693	σ_year_ = 0.753 σ_Seal ID_ = 0.103
CNS_plasma_ ~ Mass + (1|Year)	45.78776	13.014	σ_year_ = 0.764 σ_Mass_ = 0.091
CNS_RBC_ ~ (1|Year)	29.58741	–	σ_year_ = 0.193
CNS_RBC_ ~ Sex + (1|Year)	32.38981	2.8024	σ_year_ = 0.210 σ_Sex_ = 0.281
CNS_RBC_ ~ Mass + (1|Year)	39.74659	10.1592	σ_year_ = 0.195 σ_Mass_ = 0.063
CNS_RBC_ ~ (1|Year/Seal ID)	107.9329	78.3455	σ_year_ = 0.178 σ_Seal ID_ = 0.134

Median variance (σ) reflects the 50% quantiles of variance for each model effect. Year and seal ID were random effects, sex was a fixed effect, and mass was a continuous effect

CNS_plasma_: plasma tissue, CNS_RBC_: red blood cell tissue

**Fig. 5:**
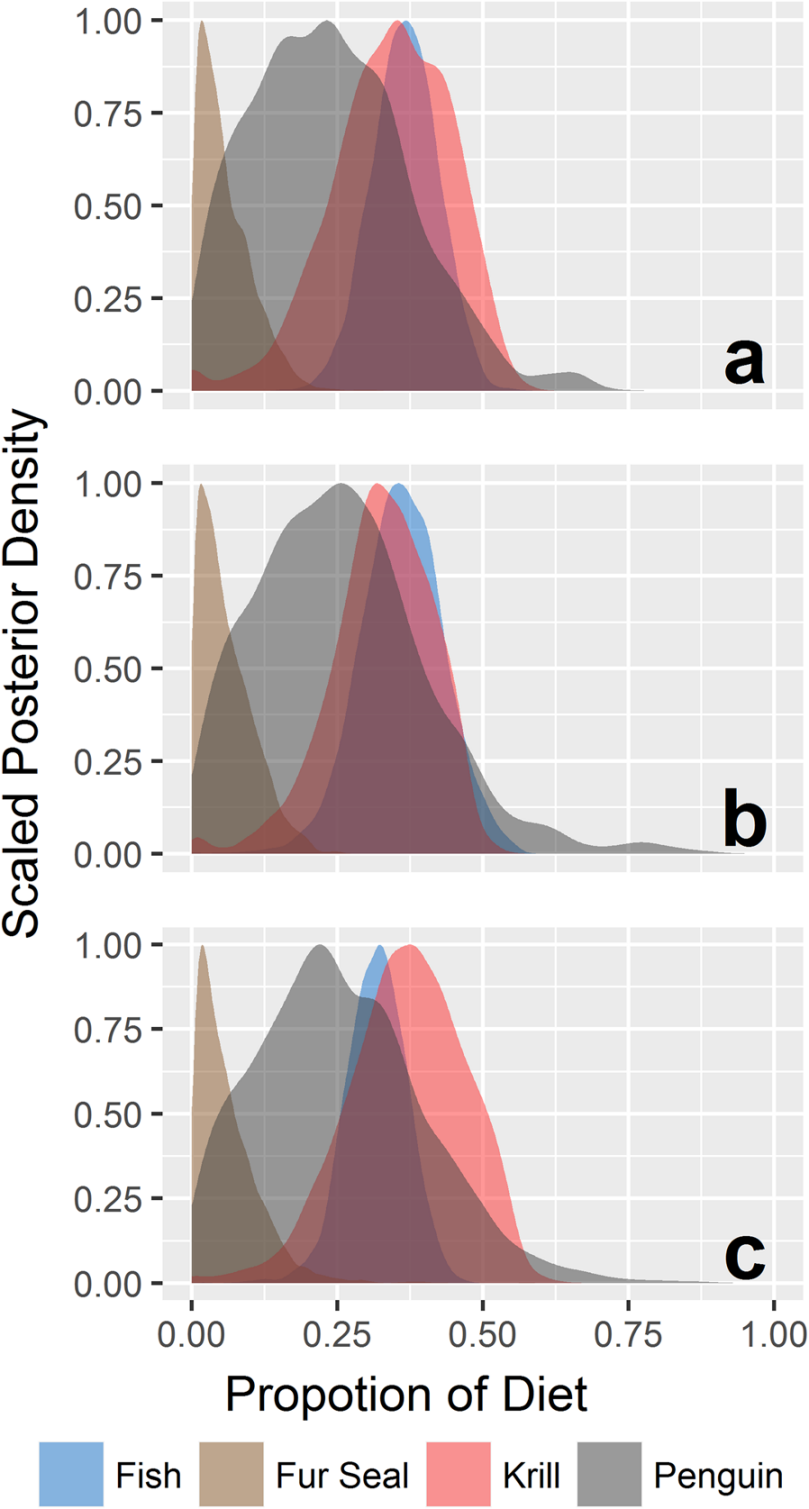
Estimates of prey contribution to the diet of leopard sealsfrom the Bayesian stable isotope mixing model posterior densities incorporating the *δ*^13^C, *δ*^15^N, and *δ*^34^S data from four prey sources and leopard seal RBCs collected in a) 2013 (*n *= 9), b) 2014 (*n *= 10), and c) 2017 (*n *= 4)

**Fig. 6:**
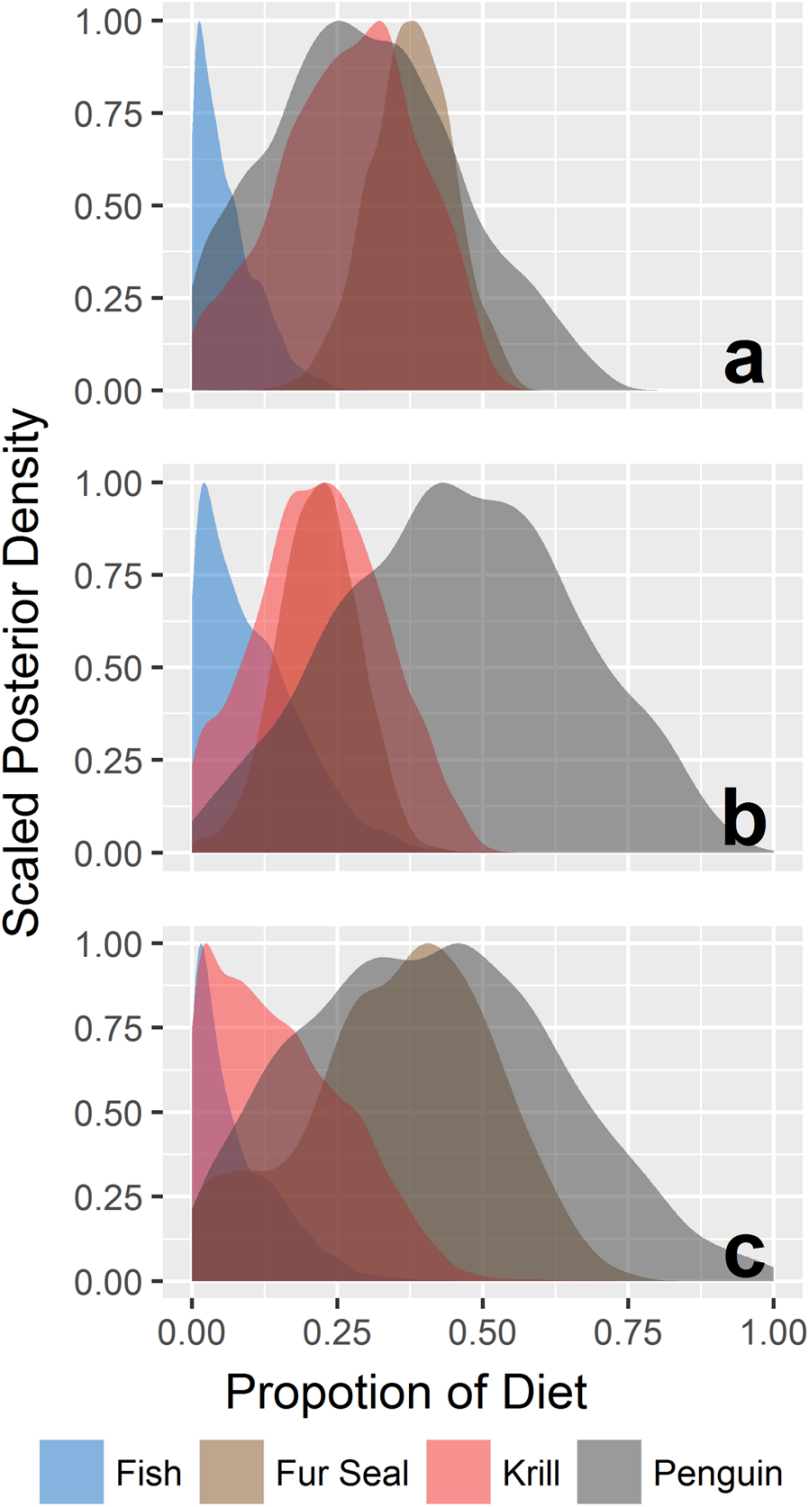
Estimates of prey contribution to the diet of leopard seals from the Bayesian stable isotope mixing model posterior densities incorporating the *δ*^13^C, *δ*^15^N, and *δ*^34^S data from four prey sources and leopard seal plasma collected in a) 2013 (*n *= 9), b) 2014 (*n *= 10) and c) 2017 (*n *= 2)

Model posterior distributions provide an estimate, with associated uncertainty, for the contributions of each prey type to the consumer diet [34]. Our posterior distributions of stable isotope values from RBCs were consistent across years (Fig. 5) and sex (Additional file 1: Table S3), but this was not the case from plasma between years (Fig. 6). Due to sex-based differences in model results, final posterior dietary estimates for plasma were conducted with males excluded (*n *= 21, Fig. 5).

### Antarctic fur seal availability as prey

**Fig. 7:**
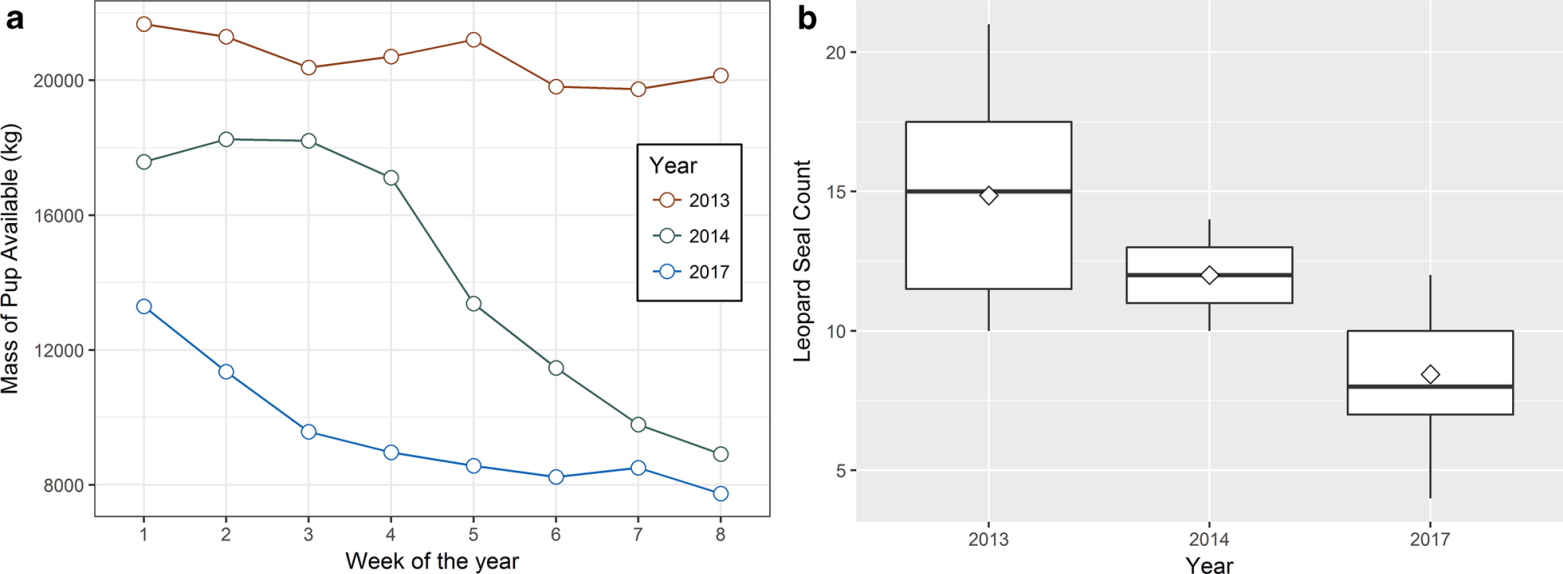
**a** Indices of Antarctic fur seal pup availability for leopard seal predation. Week 1 is the first week of January. **b** Weekly census counts of adult female leopard seals hauled out at Cape Shirreff during January to March for 2013, 2014, and 2017. Boxes indicate median and upper and lower 75th and 25th percentiles; whiskers represent 10th and 90th percentiles, and open diamonds indicate mean values

When pup growth and weekly counts are taken into account, fur seal pups were less available as a prey resource to leopard seals in each successive year of the study (Fig. 7a). The drop in pup availability during the 2014 season was particularly steep. The number of adult female leopard seals counted in the weekly census during January through March were similar between 2013 and 2014, but markedly lower in 2017 (Fig. 7b).

## Discussion

Our a priori observations identified four groups of leopard seal prey items (fish, fur seal, krill, and penguin) which were well supported by our stable isotope data (Fig. 2).

### Weekly diet variability

As the KNN comparison results for the *δ*^13^C/*δ*^15^N/*δ*^34^S values from plasma indicated no differences between capture dates, we conclude there were no substantial differences in leopard seal diets over weekly time scales during summer. This inference is supported by previous, land-based, opportunistic observations of individuals repeatedly foraging at one location on particular prey over these time scales [15, 20].

### Seasonal diet variability

Leopard seals were typically sampled within 1 week of arrival to Cape Shirreff (December through February), and RBCs reflect seal diets from about 1 month prior and up to collection. Therefore, the dietary estimations from MixSIAR using stable isotope data from the seals’ RBCs reflect foraging behavior in the early austral spring/summer months (~ November through January). These pre-arrival diets were consistent across all years (Fig. 5), and between sexes (Additional file 1: Table S3). They were characterized by krill (31.7–38.0%), fish (31.6–36.5%), and penguin (24.4–26.9%). Such consistency suggests that, irrespective of sex, leopard seals in the western Antarctic Peninsula (WAP) depend largely upon krill and fish during the spring months when mesopredator prey are more broadly distributed and, therefore, less available.

Leopard seal diets changed quickly, however, after arriving to Cape Shirreff. The *δ*^15^N values from plasma, which reflect seal diet from about 1 week prior to collection, from females were significantly higher (average of +2.1‰) than those from RBCs, representing a potential increase of about half a trophic level within a week of arrival. Previous work demonstrated that the *δ*^15^N values from plasma from captive northern fur seals (*Callorhinus urinus*) consuming known diets were an average of 1.1‰ higher than those from RBCs, likely due to differences in their amino acid composition [50]. However, the trend of higher *δ*^15^N values from plasma collected from female seals we observed is larger than 1.1‰ and consistent across all observations, suggesting a diet change likely driven by increased consumption of fur seal pups by females. The *δ*^15^N values from plasma for males only differed from those of their RBCs by a mean of 0.5‰, which may or may not be indicative of a diet switch.

This differential prey selection by sex is also evident from the MixSIAR results for the CNS model using isotope values from plasma (Additional file 1: Table S3). However, inference based solely upon CNS model results should be made with caution given our moderate sample sizes. While much of the sampling-associated uncertainty is explicitly propagated through the Bayesian framework, results based on small samples, like male leopard seal tissues (N = 2), require additional context. In this case, several other lines of evidence support sex-based differences in leopard seal diets during early austral summer. Leopard seals are sexually dimorphic and the larger, territorial females exclude males and less dominant females from foraging near fur seal colonies [21, 22]. While we record female leopard seals daily, even hourly, hunting near fur seal beaches at Cape Shirreff during the summer, since 2011 we observed only a single male doing so (U.S. AMLR unpublished data). During 9 seasons of observing leopard seal kills at Seal Island, when sex was observable, 100% of fur seal pups were taken by adult females [20]. Finally, records spanning the Antarctic and sub-Antarctic across decades, indicate sex-based differences in foraging and migration patterns likely result in differential diet (summarized by [15]).

The trend of female leopard seals consuming more fur seals than males may be related to the energetic demands of breeding. Cape Shirreff is the largest fur seal breeding colony in the WAP [82]. Leopard seals are capital breeders that likely wean their pups in December [10] and reach peak molt in early February (U.S. AMLR unpublished data). Therefore, December-February is a brief window during which leopard seals must consume adequate resources to compensate for these two energetically demanding events [83]. The unusually-dense summer aggregations of leopard seals at Cape Shirreff, then, are possibly driven by the availability of easily-consumed, energy-rich Antarctic fur seal pups.

In addition to such sex-based differences in life history, the foraging behavior of leopard seals is influenced by their body size. In high density foraging environments, like Cape Shirreff, adult female leopard seals compete intraspecifically in ways beyond excluding males [22, 84]. For example, larger females regularly kleptoparasitize fur seal pups from their smaller conspecifics [21]. Additionally, leopard seals maximize fur seal and penguin harvesting by caching carcasses, potentially adjusting their caching strategy based on their body size [85]. Likely due to such size-based trophic interactions, larger animals have higher *δ*^15^N values at a predictable rate (Fig. 4) that reflect their increased consumption of prey with higher *δ*^15^N values (fur seals or fish). This diet-mass relationship is present either with or without males included in the analyses. Therefore, although mass and sex effects are confounded to some degree, both appear integral to understanding leopard seal diet.

### Interannual diet variability

Summer dietary estimates from leopard seal plasma showed pronounced interannual differences (Fig. 6), and sex and year emerged as the most informative factors for mixing models. While seal ID and mass were informative co-variates, median posterior variances suggest that year and sex explained much more of the model variation (Table 4). The summer diets of adult female leopard seals were dominated by fur seal (21.3–37.5%) and penguin (29.5–46.2%) in all years, with substantial proportions of krill (21.9–26.8%) in 2013 and 2014.

Such interannual variation in the selection of fur seal pups suggests that leopard seals adjust foraging strategies on seasonal and interannual time scales in response to prey availability. Historical observations indicate that adult female leopard seals in the WAP met their December-February energetic needs by consuming recently-weaned crabeater seal (*Lobodon carcinophagus*) pups [86]. However, rapid regional warming in recent decades has removed sea-ice habitat for breeding crabeater seals [58], thereby disrupting the summer foraging patterns of leopard seals. Such disruptions can drive marine apex predators to switch to the next most energy-dense prey available [87, 88]. Hundreds of female leopard seals in the WAP have likely switched their diet focus to Antarctic fur seals at Cape Shirreff during the last two decades due to decreased availability of sea-ice dependent crabeater seals.

Before 1996 no more than two leopard seals were seen foraging concurrently at Cape Shirreff ([89, 90], D. Torres *Pers. Comm.* in [91]), but their numbers rose rapidly between 1998 and 2011 [92]. Concomitantly, after over 50 years of strong population growth, the Antarctic fur seal population at Cape Shirreff has decreased since 2007 on average 16.3% per year as measured by pup production. This significant decrease may be driven by consumption of fur seal pups by leopard seals given the number of animals present (Fig. 7b) and the proportion of fur seal in their diet (21.3 to 37.6%). Fur seal pups have become less available to leopard seals each successive year (Fig. 7a), and the proportion of fur seal in leopard seal diets decreased from 2013 to 2014 (Fig. 6). However, that proportion rebounded in 2017, coincident with a ˃ 40% decrease in the number of adult female leopard seals (Fig. 7b). The availability of Antarctic fur seals as a prey resource potentially drew leopard seals to Cape Shirreff, and as that resource has declined, it appears to have driven a decrease in leopard seal abundance as well. This interaction underscores the important role of top predators in shaping community structure and driving population trajectories of other species within their food web [1, 93, 94]. In addition, as seen in this example, anthropogenic or other perturbations to ecosystems, such as those from sea ice reduction due to climate change, can have unexpected and often drastic indirect effects across multiple levels within an ecosystem.

### Diets of leopard seals estimated across methods

Our a priori diet observations indicated that fur seal, penguin, fish, and krill, in order of highest to lowest occurrence, were the primary components of leopard seal diets (Table 2) at Cape Shirreff during the austral summer. Behavioral reports from Cape Shirreff also emphasized fur seal as a main diet component, along with penguin and fish [21, 62]. However, the dietary estimates derived from the stable isotope mixing models identify krill as a key diet component of leopard seal diets in spring and summer for both sexes. This finding supports the designation of leopard seals as a krill dependent predator [12], and emphasizes the potential bias in diet studies based solely upon scat data [17].

Krill is under-represented in leopard seal scats which may be the result of foraging patterns coupled with an overlap in their average foraging trip durations (10.3–46.6 h, [22]) and gut passage times (15.3–164 h, [95]). A recent summary of leopard seal diving behavior at Cape Shirreff reported consistent foraging patterns with high activity at night, peaks during the crepuscular hours, and resting haul-outs centered around local noon [22]. Leopard seals likely feed on krill at night, when zooplankton typically migrate toward the surface, whereas pup and penguin hunting happens mostly in daylight [21]. Different prey types move at different speeds through the intestines of leopard seals [95]. Therefore, this foraging pattern may result in the krill consumed at night being evacuated at sea, whereas mesopredator prey captured during the day are defecated on study beaches when the seals haul-out. The successful management of an increasing krill fishery in Antarctic waters relies on accurate estimates of krill occurrence in the diets of Antarctic predators which are likely impossible using estimates from scat data alone. This is especially important as over-fishing krill could result in further decreases in prey availability for leopard seals already coping with reductions in important prey from sea-ice loss and declining fur seal numbers.

## Conclusions

We integrated scat, visual observation, and stable isotope data to identify Antarctic fur seal pups, penguins, krill, and demersal notothen fishes as key prey items for leopard seals foraging in the South Shetland Islands. We aligned sample tissue turnover rates with seasonal transition periods, to illustrate leopard seal diet variability on weekly, monthly, and inter-annual time scales. The austral spring diets of males and females were consistent across all 3 years and focused on krill, fish, and to a lesser extent, penguin. The transition to summer foraging was distinct for males and females. Male diets were likely unchanged, whereas female diets transitioned rapidly to include more energy-dense Antarctic fur seal pups along with penguins. The extent of trophic enrichment, and fur seal in the diet, appears governed by a combination of leopard seal sex and body size.

Resolving the preferred prey items of leopard seals is central to understanding food web dynamics in the rapidly warming Antarctic Peninsula region [60]. This is particularly true for large adult females, which are important predators of fur seals and penguins [19–21, 62], and therefore may be affecting population dynamics of these mesopredators [88] or ecosystem function through top-down forcing [3]. In addition, climatic variability can lead to high variance in predator diets (e.g., [96]), and displaced predators may destabilize prey populations [1, 97]. Leopard seal diets and the associated predator–prey population dynamics appear to illustrate both. The rapid increase [21, 81] and subsequent decrease of leopard seals at Cape Shirreff appear to be moderated by the availability of sea ice as habitat for pagophilic crabeater seals resulting in a prey shift to fur seal pups.

Finally, as the krill fishery in the Antarctic expands [98], a full understanding of krill as a prey resource for multiple consumers in the region is essential for effective fishery management. The sex- and season-specific dietary proportions reported here will improve spatio-temporal ecosystem models that inform management of Antarctic resources (e.g., [99]). They will also increase the accuracy of bioenergetic models and deepen our understanding of Antarctic ecosystem function. Future studies should consider addressing longer time scales by analyzing the stable isotope values from slow-growing tissues like vibrissae or teeth.

## Supplementary information

**Additional file 1.** Four data and analysis tables providing mean isotope values for prey sources, mean isotope values for consumer tissues, model-estimated leopard seal dietary proportions, and raw isotope data.

## Data Availability

All data generated or analysed during this study are included in this published article and its Additional files.
