# Supplementary material for: Leopard seal diets in a rapidly warming polar region vary by year, season, sex, and body size
Source: BMC Ecol. 2020 Jun 3;20:32. doi: 10.1186/s12898-020-00300-y (PMC7271520; doi:10.1186/s12898-020-00300-y)
Supplement: Supplementary file 1 — Additional file 1. Four data and analysis tables providing mean isotope values for prey sources, mean isotope values for consumer tissues, model-estimated leopard seal dietary proportions, and raw isotope data. [file 12898_2020_300_MOESM1_ESM.docx]

Supplementary Materials. D.J. Krause, M.E. Goebel, C. Kurle. Leopard seal diets in a rapidly warming polar region vary by year, season, sex, and body size. BMC Ecology.

Table S1. The mean (±SD; ‰) *δ*^13^C, *δ*^15^N, and *δ*^34^S values for all leopard seal prey sources grouped by year. Species: *Notothenia coriiceps* (Nc)*, Trematomus newnesi* (Tn), *Arctocephalus gazella* (Ag), *Euphausia superba* (Es), *Pygoscelis papua* (Pp), *Pygoscelis antarcticus* (Pa).

| Prey | Year | *n* | Species | Size/Life Stage | δ^13^C (‰) | δ^15^N (‰) | δ^34^S (‰) |
| --- | --- | --- | --- | --- | --- | --- | --- |
| Fish | 2014 | 6 | Nc, Tn | Adult/>40 cm | -23.0 ± 1.6 | 11.9 ± 1.1 | 18.0 ± 0.5 |
|  | 2017 | 3 | Nc | Adult/>40 cm | -21.1 ± 0.6 | 12.1 ± 0.6 | 18.1 ± 0.5 |
| Fur seal | 2013 | 3 | Ag | Pup | -22.8 ± 0.8 | 12.8 ± 0.3 | 18.7 ± 0.4 |
|  | 2014 | 12 | Ag | Pup | -22.1 ± 1.5 | 12.6 ± 0.8 | 19.2 ± 0.4 |
|  | 2017 | 7 | Ag | Pup | -23.7 ± 0.6 | 11.8 ± 0.4 | 18.4 ± 0.4 |
| Krill | 2013 | 10 | Es | Adult/43-54 mm | -27.4 ± 0.8 | 5.1 ± 0.3 | 19.0 ± 0.4 |
|  | 2014 | 10 | Es | Adult/41-50 mm | -28.6 ± 0.6 | 4.2 ± 0.5 | 19.7 ± 0.2 |
|  | 2017 | 10 | Es | Adult/46-55 mm | -27.5 ± 0.7 | 4.2 ± 0.7 | 18.8 ± 0.5 |
| Penguin^1^ | 2013 | 8 | Pp, Pa | Adult, Chick | -26.6 ± 0.5 | 7.4 ± 0.9 | 18.8 ± 0.4 |
|  | 2014 | 15 | Pp, Pa | Adult, Chick | -26.1 ± 0.6 | 7.9 ± 0.9 | 18.9 ± 0.4 |

^1^ The *δ*^13^C, *δ*^15^N, and *δ*^34^S values for gentoo and chinstrap penguins were not compatible

with rejection of H_o_ (no spatial difference) and were therefore combined; KNN, P=0.89.

Table S2. The mean (±SD; ‰) *δ*^13^C, *δ*^15^N, and *δ*^34^S values for leopard seal tissues grouped by year and capture. KNN lists results of K nearest-neighbors randomization tests within years for a given tissue. The null hypothesis was no spatial overlap between captures.

| Tissue | Year | Capture | *n* | δ^13^C (‰) | δ^15^N (‰) | δ^34^S (‰) | KNN |
| --- | --- | --- | --- | --- | --- | --- | --- |
| Plasma | 2013 | 1 | 9 | -24.0 ± 0.4 | 12.9 ± 0.5 | 18.4 ± 0.1 | P > 0.99 |
|  |  | 2 | 6 | -23.9 ± 0.4 | 13.0 ± 0.6 | 18.4 ± 0.1 |  |
|  | 2014 | 1 | 10 | -24.5 ± 0.4 | 12.6 ± 0.8 | 18.4 ± 0.1 | P > 0.99 |
|  |  | 2 | 9 | -24.4 ± 0.6 | 12.9 ± 0.5 | 18.4 ± 0.2 |  |
|  | 2017 | 1 | 4 | -24.0 ± 0.2 | 12.1 ± 1.4 | 18.6 ± 0.2 | P > 0.99 |
|  |  | 2 | 3 | -24.0 ± 0.1 | 12.9 ± 0.9 | 18.6 ± 0.2 |  |
| Red blood cells | 2013 | 1 | 9 | -23.3 ± 0.2 | 11.1 ± 0.6 | 17.7 ± 0.4 | P > 0.99 |
|  |  | 2 | 6 | -23.4 ± 0.3 | 11.4 ± 0.4 | 17.7 ± 0.3 |  |
|  | 2014 | 1 | 10 | -23.9 ± 0.4 | 11.0 ± 0.7 | 18.1 ± 0.2 | P > 0.99 |
|  |  | 2 | 9 | -23.8 ± 0.3 | 11.2 ± 0.4 | 18.1 ± 0.3 |  |
|  | 2017 | 1 | 4 | -23.5 ± 0.3 | 10.7 ± 0.4 | 18.5 ± 0.1 | P > 0.99 |
|  |  | 2 | 3 | -23.4 ± 0.2 | 10.7 ± 0.8 | 18.3 ± 0.4 |  |

Table S3. The estimated mean proportions, with one standard deviation (SD) and credible intervals (CI), of estimated prey sources (%) in diets of female (♀) and male (♂) leopard seals for each of three years (2013, 2014, and 2017) as determined by a Bayesian stable isotope mixing model (MixSIAR) using the *δ*^13^C, *δ*^15^N, and *δ*^34^S values from seal plasma tissue (“Plasma”) and red blood cells (“RBC”) based on four prey categories (fish, fur seal, krill, and penguin).

Plasma RBC

|  |  | Mean | SD | 2.5% CI | 50% CI | 97.5% CI |  | Mean | SD | 2.5% CI | 50% CI | 97.5% CI |
| --- | --- | --- | --- | --- | --- | --- | --- | --- | --- | --- | --- | --- |
| 2013 ♀ | Fish | 6.0 | 5.3 | 0.1 | 4.6 | 18.9 |  | 36.5 | 5.8 | 24.8 | 36.7 | 47.6 |
|  | Fur seal | 37.6 | 7.3 | 23.0 | 37.7 | 51.6 |  | 5.5 | 4.5 | 0.2 | 4.4 | 16.8 |
|  | Krill | 26.8 | 12.2 | 2.1 | 27.4 | 48.1 |  | 34.1 | 10.0 | 12.0 | 34.7 | 50.7 |
|  | Penguin | 29.5 | 16.7 | 0.6 | 29.0 | 62.5 |  | 23.9 | 13.1 | 2.1 | 23.1 | 51.8 |

| 2014 ♀ | Fish | 10.6 | 9.0 | 0.1 | 8.7 | 32.5 |  | 36.0 | 7.1 | 22.0 | 36.0 | 49.8 |
| --- | --- | --- | --- | --- | --- | --- | --- | --- | --- | --- | --- | --- |
|  | Fur seal | 21.3 | 7.1 | 6.5 | 21.3 | 35.1 |  | 5.7 | 4.6 | 0.2 | 4.7 | 16.8 |
|  | Krill | 21.9 | 11.5 | 0.5 | 21.7 | 44.3 |  | 32.4 | 8.5 | 14.2 | 33.1 | 46.7 |
|  | Penguin | 46.2 | 21.1 | 5.1 | 46.7 | 85.2 |  | 25.9 | 14.2 | 2.4 | 24.8 | 56.6 |

| 2017 ♀ | Fish | 8.0 | 7.7 | 0.1 | 5.3 | 26.6 |  | 31.7 | 5.2 | 21.5 | 31.7 | 41.4 |
| --- | --- | --- | --- | --- | --- | --- | --- | --- | --- | --- | --- | --- |
|  | Fur seal | 34.2 | 16.8 | 0.6 | 35.3 | 64.2 |  | 6.0 | 5.1 | 0.2 | 4.6 | 18.5 |
|  | Krill | 15.4 | 11.8 | 0.1 | 13.5 | 40.7 |  | 36.6 | 10.1 | 15.0 | 37.1 | 54.2 |
|  | Penguin | 42.4 | 22.3 | 2.3 | 42.0 | 84.9 |  | 25.8 | 14.1 | 2.5 | 24.9 | 56.3 |

| 2017 ♂ | Fish | 11.7 | 11.6 | 0.1 | 8.8 | 37.2 |  | 31.6 | 6.8 | 17.5 | 32.0 | 43.8 |
| --- | --- | --- | --- | --- | --- | --- | --- | --- | --- | --- | --- | --- |
|  | Fur seal | 11.8 | 14.1 | 0.1 | 5.4 | 45.4 |  | 6.0 | 6.8 | 0.01 | 3.9 | 23.4 |
|  | Krill | 26.9 | 24.6 | 0.1 | 25.5 | 66.1 |  | 38.0 | 12.2 | 9.6 | 39.0 | 57.9 |
|  | Penguin | 49.5 | 38.5 | 0.1 | 47.1 | 99.0 |  | 24.4 | 16.7 | 0.02 | 22.5 | 64.3 |

Table S4. Raw *δ*^13^C, *δ*^15^N, and *δ*^34^S values for leopard seal tissues grouped by year, tissue, capture, seal ID, sex and mass.

| d13C | d15N | d34S | Year | Tissue | Capture | ID | Sex | Mass |
| --- | --- | --- | --- | --- | --- | --- | --- | --- |
| -24.19 | 12.81 | 18.3 | 2013 | Plasma | 1 | 406Y | F | 462 |
| -23.84 | 13.59 | 18.3 | 2013 | Plasma | 2 | 406Y | F | 498 |
| -23.63 | 12.17 | 18.4 | 2013 | Plasma | 1 | 422Y | F | 416 |
| -23.39 | 13.09 | 18.4 | 2013 | Plasma | 1 | 12OR | F | 465 |
| -23.31 | 13.14 | 18.4 | 2013 | Plasma | 2 | 12OR | F | 446 |
| -24.1 | 13.24 | 18.3 | 2013 | Plasma | 1 | 62OR | F | 411 |
| -24.42 | 12.78 | 18.3 | 2013 | Plasma | 2 | 62OR | F | 422 |
| -23.9 | 12.81 | 18.6 | 2013 | Plasma | 1 | 394Y | F | 419 |
| -23.9 | 11.97 | 18.5 | 2013 | Plasma | 2 | 394Y | F | 416 |
| -24.43 | 12.29 | 18.5 | 2013 | Plasma | 1 | 37OR | F | 409 |
| -24.18 | 12.89 | 18.2 | 2013 | Plasma | 1 | 36OR | F | 442 |
| -24.1 | 13.06 | 18.4 | 2013 | Plasma | 2 | 36OR | F | 437 |
| -23.62 | 13.56 | 18.4 | 2013 | Plasma | 1 | 71OR | F | 446 |
| -23.62 | 13.63 | 18.5 | 2013 | Plasma | 2 | 71OR | F | 460 |
| -24.24 | 13.49 | 18.6 | 2013 | Plasma | 1 | 70OR | F | 421 |
| -24.27 | 11.92 | 18.5 | 2014 | Plasma | 1 | 63OR | F | 418 |
| -23.91 | 12.48 | 18.6 | 2014 | Plasma | 2 | 63OR | F | 432 |
| -25.04 | 11.79 | 18.2 | 2014 | Plasma | 1 | 84OR | F | 334 |
| -25.06 | 12.17 | 18.4 | 2014 | Plasma | 2 | 84OR | F | 371 |
| -24.74 | 13.05 | 18.4 | 2014 | Plasma | 1 | 9OR | F | 407 |
| -24.76 | 13.21 | 18.4 | 2014 | Plasma | 2 | 9OR | F | 414 |
| -24.58 | 13.27 | 18.4 | 2014 | Plasma | 1 | 16OR | F | 477 |
| -24.25 | 13.27 | 17.8 | 2014 | Plasma | 2 | 16OR | F | 494 |
| -23.91 | 13.26 | 18.4 | 2014 | Plasma | 1 | 18OR | F | 492 |
| -23.88 | 13.29 | 18.5 | 2014 | Plasma | 2 | 18OR | F | 494 |
| -24.47 | 12.64 | 18.6 | 2014 | Plasma | 1 | 37OR | F | 423 |
| -24.85 | 12.53 | 18.5 | 2014 | Plasma | 2 | 37OR | F | 406 |
| -25.09 | 12.71 | 18.4 | 2014 | Plasma | 1 | 397G | F | 385 |
| -24.88 | 12.88 | 18.3 | 2014 | Plasma | 2 | 397G | F | 385 |
| -23.85 | 12.35 | 18.4 | 2014 | Plasma | 1 | 401Y | F | 497 |
| -23.41 | 12.26 | 18.5 | 2014 | Plasma | 2 | 401Y | F | 485 |
| -24.37 | 13.62 | 18.4 | 2014 | Plasma | 1 | 406Y | F | 501 |
| -24.23 | 13.67 | 18.3 | 2014 | Plasma | 2 | 406Y | F | 533 |
| -24.55 | 10.97 | 18.3 | 2014 | Plasma | 1 | 58OR | F | 409 |
| -24.13 | 13.3 | 18.7 | 2017 | Plasma | 1 | 84OR | F | 437 |
| -24.12 | 13.34 | 18.6 | 2017 | Plasma | 2 | 84OR | F | 437 |
| -23.75 | 11.08 | 18.4 | 2017 | Plasma | 2 | 120OR | M | 329 |
| -24.24 | 10.73 | 18.4 | 2017 | Plasma | 1 | 111OR | M | 261 |
| -23.88 | 11.78 | 18.4 | 2017 | Plasma | 2 | 111OR | M | 251 |
| -23.91 | 13.26 | 18.7 | 2017 | Plasma | 1 | 12OR | F | 537 |
| -23.99 | 13.45 | 18.7 | 2017 | Plasma | 2 | 12OR | F | 543 |
| -23.09 | 11.57 | 17.1 | 2013 | RBC | 1 | 406Y | F | 462 |
| -23.99 | 11.98 | 17.3 | 2013 | RBC | 2 | 406Y | F | 498 |
| -23.45 | 10.43 | 17.5 | 2013 | RBC | 1 | 422Y | F | 416 |
| -23.15 | 10.85 | 17.5 | 2013 | RBC | 1 | 12OR | F | 465 |
| -23.15 | 11.32 | 17.4 | 2013 | RBC | 2 | 12OR | F | 446 |
| -23.21 | 11.65 | 17.7 | 2013 | RBC | 1 | 62OR | F | 411 |
| -23.36 | 11.85 | 17.6 | 2013 | RBC | 2 | 62OR | F | 422 |
| -23.24 | 11.25 | 17.8 | 2013 | RBC | 1 | 394Y | F | 419 |
| -23.27 | 11.07 | 17.8 | 2013 | RBC | 2 | 394Y | F | 416 |
| -23.83 | 10.28 | 18 | 2013 | RBC | 1 | 37OR | F | 409 |
| -23.38 | 10.68 | 17.9 | 2013 | RBC | 1 | 36OR | F | 442 |
| -23.53 | 11.09 | 17.7 | 2013 | RBC | 2 | 36OR | F | 437 |
| -23.3 | 10.59 | 17.4 | 2013 | RBC | 1 | 71OR | F | 446 |
| -23.25 | 11 | 18.1 | 2013 | RBC | 2 | 71OR | F | 460 |
| -23.34 | 12.14 | 18.4 | 2013 | RBC | 1 | 70OR | F | 421 |
| -23.88 | 10.74 | 18.1 | 2014 | RBC | 1 | 63OR | F | 418 |
| -23.79 | 11.13 | 18.3 | 2014 | RBC | 2 | 63OR | F | 432 |
| -24.35 | 10.51 | 18.2 | 2014 | RBC | 1 | 84OR | F | 334 |
| -24.24 | 10.66 | 18.2 | 2014 | RBC | 2 | 84OR | F | 371 |
| -24.31 | 11.62 | 18.1 | 2014 | RBC | 1 | 9OR | F | 407 |
| -24.14 | 11.56 | 17.7 | 2014 | RBC | 2 | 9OR | F | 414 |
| -23.92 | 11.8 | 18 | 2014 | RBC | 1 | 16OR | F | 477 |
| -23.55 | 11.46 | 18.2 | 2014 | RBC | 2 | 16OR | F | 494 |
| -23.75 | 10.04 | 18.2 | 2014 | RBC | 1 | 18OR | F | 492 |
| -23.59 | 10.73 | 18.4 | 2014 | RBC | 2 | 18OR | F | 494 |
| -24.22 | 10.76 | 17.7 | 2014 | RBC | 1 | 37OR | F | 423 |
| -23.92 | 10.49 | 18.1 | 2014 | RBC | 2 | 37OR | F | 406 |
| -23.97 | 10.61 | 18.2 | 2014 | RBC | 1 | 397G | F | 385 |
| -24.17 | 11.52 | 18.4 | 2014 | RBC | 2 | 397G | F | 385 |
| -23.43 | 11.84 | 18.4 | 2014 | RBC | 1 | 401Y | F | 497 |
| -23.4 | 11.39 | 18.3 | 2014 | RBC | 2 | 401Y | F | 485 |
| -23.6 | 11.84 | 18.1 | 2014 | RBC | 1 | 406Y | F | 501 |
| -23.51 | 11.66 | 17.7 | 2014 | RBC | 2 | 406Y | F | 533 |
| -23.31 | 10.55 | 17.9 | 2014 | RBC | 1 | 58OR | F | 409 |
| -23.72 | 11.01 | 18.6 | 2017 | RBC | 1 | 84OR | F | 437 |
| -23.59 | 11.1 | 17.9 | 2017 | RBC | 2 | 84OR | F | 437 |
| -23.61 | 10.35 | 18.4 | 2017 | RBC | 1 | 120OR | M | 329 |
| -23.09 | 10.39 | 18.4 | 2017 | RBC | 1 | 111OR | M | 261 |
| -23.54 | 9.71 | 18.4 | 2017 | RBC | 2 | 111OR | M | 251 |
| -23.39 | 10.97 | 18.6 | 2017 | RBC | 1 | 12OR | F | 537 |
| -23.16 | 11.2 | 18.6 | 2017 | RBC | 2 | 12OR | F | 543 |
